# Speech-Based Parkinson’s Detection Using Pre-Trained Self-Supervised Automatic Speech Recognition (ASR) Models and Supervised Contrastive Learning

**DOI:** 10.3390/bioengineering12070728

**Published:** 2025-07-01

**Authors:** Hadi Sedigh Malekroodi, Nuwan Madusanka, Byeong-il Lee, Myunggi Yi

**Affiliations:** 1 Industry 4.0 Convergence Bionics Engineering, Pukyong National University, Busan 48513, Republic of Korea; hadi_sedigh@pukyong.ac.kr (H.S.M.); bilee@pknu.ac.kr (B.-i.L.); 2 Digital Healthcare Research Center, Institute of Information Technology and Convergence, Pukyong National University, Busan 48513, Republic of Korea; nuwanmadusanka@hotmail.com; 3 Major of Human Bioconvergence, Division of Smart Healthcare, Pukyong National University, Busan 48513, Republic of Korea; 4 Major of Biomedical Engineering, Division of Smart Healthcare, Pukyong National University, Busan 48513, Republic of Korea

**Keywords:** Parkinson’s disease (PD), deep learning, transfer learning, supervised contrastive learning, Wav2Vec 2.0, HuBERT

## Abstract

Diagnosing Parkinson’s disease (PD) through speech analysis is a promising area of research, as speech impairments are often one of the early signs of the disease. This study investigates the efficacy of fine-tuning pre-trained Automatic Speech Recognition (ASR) models, specifically Wav2Vec 2.0 and HuBERT, for PD detection using transfer learning. These models, pre-trained on large unlabeled datasets, can be capable of learning rich speech representations that capture acoustic markers of PD. The study also proposes the integration of a supervised contrastive (SupCon) learning approach to enhance the models’ ability to distinguish PD-specific features. Additionally, the proposed ASR-based features were compared against two common acoustic feature sets: mel-frequency cepstral coefficients (MFCCs) and the extended Geneva minimalistic acoustic parameter set (eGeMAPS) as a baseline. We also employed a gradient-based method, Grad-CAM, to visualize important speech regions contributing to the models’ predictions. The experiments, conducted using the NeuroVoz dataset, demonstrated that features extracted from the pre-trained ASR models exhibited superior performance compared to the baseline features. The results also reveal that the method integrating SupCon consistently outperforms traditional cross-entropy (CE)-based models. Wav2Vec 2.0 and HuBERT with SupCon achieved the highest F1 scores of 90.0% and 88.99%, respectively. Additionally, their AUC scores in the ROC analysis surpassed those of the CE models, which had comparatively lower AUCs, ranging from 0.84 to 0.89. These results highlight the potential of ASR-based models as scalable, non-invasive tools for diagnosing and monitoring PD, offering a promising avenue for the early detection and management of this debilitating condition.

## 1. Introduction

Parkinson’s disease (PD), a progressive neurodegenerative disorder, affects millions of people worldwide, causing significant motor, cognitive, and speech impairments [1,2]. With the global prevalence of PD projected to double by 2040, accurate and timely diagnosis and the monitoring of disease progression have become crucial in optimizing treatment outcomes and improving patients’ quality of life [3,4,5]. Traditional assessment methods, such as the Unified PD Rating Scale (UPDRS), rely on subjective clinical evaluations, which can be time-consuming, costly, and prone to inter-rater variability [6,7].

Speech impairments in PD manifest across multiple dimensions and are among the earliest detectable symptoms [8]. Key manifestations include phonatory impairments (reduced vocal intensity, altered fundamental frequency, increased jitter and shimmer), articulatory deficits (imprecise consonant production, a reduced range of motion), prosodic alterations (monotone speech, reduced stress contrasts, altered intonation), and temporal disruptions (an altered speech rate, disrupted timing patterns) [3,8,9]. These speech changes offer valuable insights for early detection and ongoing assessment [10].

Advancements in signal processing, machine learning (ML), and deep learning (DL) have facilitated the automated detection and classification of PD severity through speech [11]. Typically, these methods employ a two-stage pipeline: feature extraction followed by classification. The development of these systems relies on supervised learning techniques, utilizing speech samples collected from both healthy individuals and those diagnosed with PD. The binary classification (Parkinson’s vs. non-Parkinson’s) is based on clinical diagnoses performed by neurologists and movement disorder specialists. Researchers have explored a wide range of models and feature extraction strategies to highlight the distinctive characteristics of PD-affected speech [2,12,13,14].

Two primary research directions have emerged based on these advancements. The first utilizes classical machine learning algorithms—including Support Vector Machines (SVMs), random forests, and k-Nearest Neighbors (kNNs)—with hand-crafted features to identify voice changes in Parkinson’s Disease patients [14,15,16,17,18,19,20]. These features include acoustic characteristics like jitter, shimmer, and harmonic-to-noise ratios, as well as complexity measurements [12,13,14,19,20]. The second direction uses deep learning architectures, which have demonstrated superior performance by automatically learning features directly from the data. Architectures such as Convolutional Neural Networks (CNNs), Recurrent Neural Networks (RNNs), and Transformer-based models have achieved state-of-the-art results in PD detection using speech [20,21,22,23,24,25,26,27,28]. These models can learn from various input forms—including raw audio, spectrograms, or a combination—without relying on manually engineered features. Moreover, transfer learning further boosts performance by leveraging pre-trained models from related speech tasks [20,29].

However, in various fields, acquiring labeled data poses significant challenges. Consequently, there has been substantial progress in pre-training deep learning models with unlabeled data. Notably, automatic speech recognition (ASR) models pre-trained on large unlabeled corpora—such as Wav2Vec 2.0 [30] and HuBERT [31]—have shown strong performance in tasks like voice pathology detection [32,33,34,35].

Originally developed for speech-to-text tasks, these models can be repurposed to detect acoustic biomarkers of disease, including those relevant to PD. For instance, HuBERT features have been found to outperform traditional acoustic features in classifying dysarthric speech, with significant improvements in both detection and severity classification accuracy [32]. Similarly, Wav2Vec 1.0 has shown robust performance, with AUROC scores ranging from 0.77 to 0.98 in cross-database PD classification [36]. Building on this, Wav2Vec 2.0 has further advanced PD detection, outperforming its predecessor across various speech modes and languages, consistently demonstrating higher accuracy in distinguishing Parkinsonian speech patterns [37]. Advanced techniques, such as layer selection and parameter-efficient fine-tuning in Wav2Vec 2.0, have achieved up to 85% accuracy on the PC-GITA dataset [38]. Additionally, another study used Wav2Vec 2.0 to classify raw speech collected from smartphones for PD detection, achieving a notable accuracy of up to 97.92%. However, challenges remain in achieving precise multi-class classification across different disease stages [39].

In this study, we first explored the potential of popular ASR models—Wav2Vec 2.0 and HuBERT—for the detection of PD based on speech using the NeuroVoz voice and speech dataset. We propose that these models, having learned rich speech representations during large-scale pre-training, can effectively capture the acoustic patterns associated with PD.

As our primary contribution, we propose a training strategy that integrates supervised contrastive (SupCon) learning [40] into a classification head—an approach that has not previously been applied in this context. Unlike previous work relying on standard cross-entropy loss, our method leverages SupCon to enhance the discriminative power of the learned representations, aiming to improve classification performance. To benchmark our deep learning approach, we also implement two classical baselines: an extreme gradient boosting (XGBoost) model [41] and a multilayer perceptron (MLP). These models were trained on traditional acoustic features: mel-frequency cepstral coefficients (MFCCs) and the extended Geneva minimalistic acoustic parameter set (eGeMAPS) [42]. Additionally, we investigate the explainability of these deep learning models. Using gradient-based methods like Grad-CAM [43], we visualize the regions of the speech signal that are most critical to their predictions.

Our evaluation, including fine-tuning and the impact of SupCon learning, indicates that this methodology enhances the accuracy of PD detection systems.

## 2. Materials and Methods

**Figure 1:**
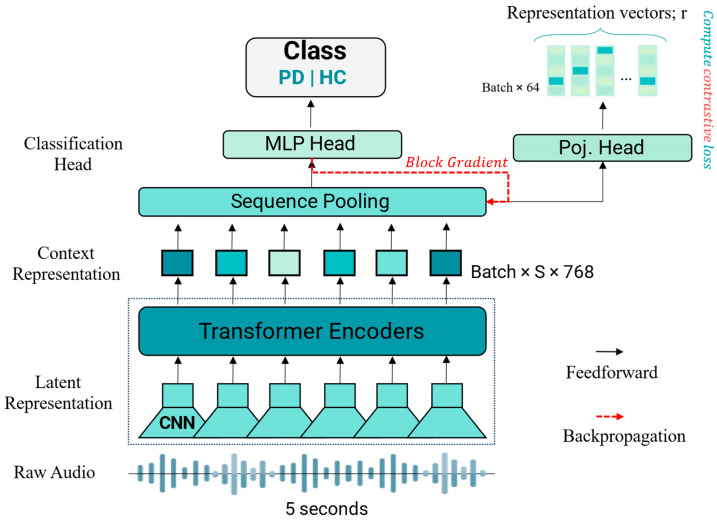
Diagram outlining the process of our classification model.

The speech classification system, depicted in Figure 1, analyzes 5 s audio clips to identify individuals with PD. The process begins with a transformer encoder that converts the raw audio into a high-dimensional feature representation. This representation is then forwarded through two parallel pathways: a classification head that predicts the label as either “Healthy” or “Parkinson”, and a projection head designed to improve model robustness through SupCon learning.

For the base model, only the classifier is utilized. To investigate the effects of SupCon learning, both the classifier and the projection network are employed, with gradient flow from the classification head blocked. The architecture employs a unified training approach where the classifier, encoder, and projection networks are trained simultaneously. By blocking the backward flow of gradients from the classifier to the encoder, this design eliminates the traditional requirement for separate training phases.

### 2.1. Dataset

This study used the recently updated NeuroVoz voice and speech database, as originally described in [44,45], which includes recordings from 112 adult native speakers of Castilian Spanish. The participants are divided into two groups: 58 healthy controls (HCs) and 54 individuals diagnosed with PD. The recordings were collected by the Bioengineering and Optoelectronics Group at the Universidad Politécnica de Madrid, in collaboration with the Otorhinolaryngology and Neurology Departments of Gregorio Marañón Hospital in Madrid, Spain. The database features a variety of speech tasks, such as sustained vowel phonation, diadochokinetic (DDK) tests, the recitation of predetermined sentences, and spontaneous speech describing an image. Further details about the database can be found in references [44,45].

In this study, we used a subset of the complete dataset, consisting of 16 repeated utterances totaling 1695 audio samples. Of these, 828 were from individuals with PD, and 867 were from HCs. This selection was made because we aimed to utilize an ASR model that had been pre-trained on a comparable large-scale dataset. The histogram in Figure S1 illustrates the distribution of audio recording durations for both the healthy control (HC) and PD groups. This highlights the inherent variability in utterance lengths within the dataset and underscores the necessity for standardizing recording durations during preprocessing. Further details about the speech tasks, including the specific sentence transcriptions with IPA and English translations (Table S1) and an overview of how these sentences map to assessments of velopharyngeal closure, prosody, and intonation-emotion (Table S2), are provided in the Supplementary Materials. To our knowledge, while one study has examined small subsets of this dataset using DDK and vowels [20], no comprehensive analysis of the complete subset has been conducted to date.

### 2.2. Data Preprocessing

To prepare the audio data for analysis by deep learning models, several preprocessing steps were implemented. The first step standardized all recordings to a 16,000 Hz sampling rate. Next, silent segments at the beginning and end of each recording were eliminated. As illustrated in Figure S1, the dataset contained recordings of varied durations. To facilitate uniform batch processing during model training, each recording was adjusted to a standard 5 s length. This was achieved by adding padding to shorter segments and cutting off excess audio from longer recordings. To enhance model generalization and reduce overfitting from the limited training data, we expanded the training set using audio augmentation. Specifically, low-pass filtering from the torch audio augmentation library [46] was applied. To ensure that the validation and test sets resembled real-world data, no augmentation was applied to them.

### 2.3. Deep Learning Models

This research evaluates the effectiveness of two leading self-supervised models, Wav2Vec 2.0 [30] and HuBERT [31], by applying a fine-tuning strategy that incorporates SupCon learning. Unlike traditional speech recognition systems, which depend on multi-stage pipelines involving hand-crafted features such as MFCCs and Hidden Markov Models (HMMs), and require significant domain expertise [47], these self-supervised models are designed to learn directly from raw audio without the need for transcribed data during pre-training. Both Wav2Vec 2.0 and HuBERT were pre-trained on the widely used LibriSpeech corpus, enabling them to capture rich speech representations.

**Figure 2:**
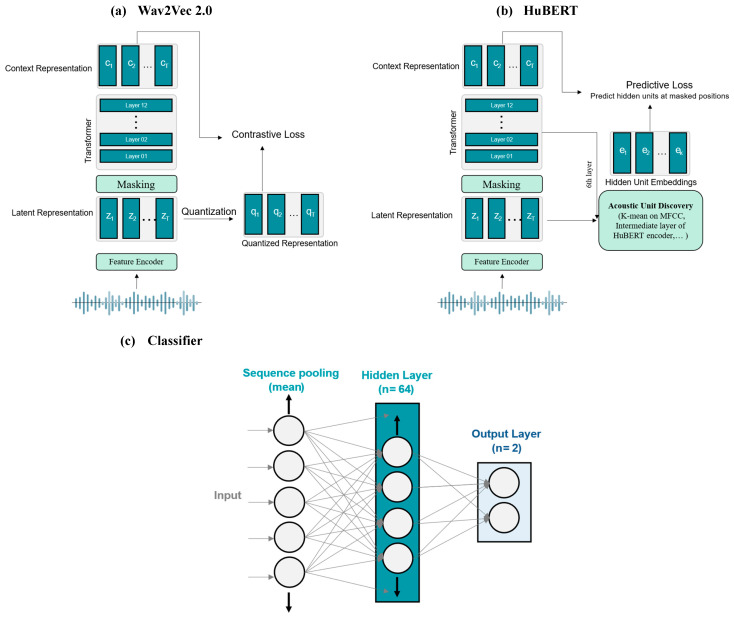
Overview of the model architecture used in this study. (**a**) Wav2Vec 2.0, (**b**) HuBERT, (**c**) classifier.

Architecturally, they leverage multilayer CNN encoders and 12-layer transformer-based context networks (Figure 2). Wav2Vec 2.0 processes raw audio into 768-dimensional contextual embeddings through convolutional layers with a stride of 20 ms and a receptive field of 25 ms, followed by masked transformer-based context modeling and a contrastive learning objective to distinguish true quantized representations from distractors, as described by Baevski et al. [30]. In contrast, HuBERT employs a k-means clustering step to generate discrete target units from MFCC features, learning to predict embeddings for masked segments using cross-entropy loss, with clustering refined iteratively using deeper model layers [31]. Both models output 768-dimensional feature vectors from their final transformer layers, which we use for analysis. For PD detection, these feature vectors are passed through a simple one-layer fully connected classifier (Figure 2c), which reduces them to 64 dimensions via a linear transformation and applies ReLU activation, dropout for regularization, and a final linear transformation to produce predictions. The same classification architecture, including a 32-dimensional hidden layer in the projection network, is used across all models.

### 2.4. Supervised Contrastive Learning

Contrastive learning is a powerful self-supervised learning technique that has gained significant attention in recent years, particularly in the fields of computer vision and natural language processing [40,48,49]. The core idea behind contrastive learning is to learn representations by comparing similar and dissimilar samples in a dataset. This approach enables models to capture meaningful features and relationships within the data without relying on explicit labels. The method is often used in self-supervised settings to learn from unlabeled data [40]. However, it can be adapted for supervised learning. In a supervised context, an anchor sample can have multiple positive samples (i.e., other samples from the same class). Since standard contrastive loss only handles a single positive per anchor, it is not directly applicable [40,50]. Therefore, the supervised contrastive loss generalizes this by being formulated to incorporate multiple positive samples.

Contrastive loss for supervised learning can be generalized to incorporate multiple positive samples. This is achieved by summing all positives for a given anchor sample, denoted as $P\left(i\right)$. As shown in Equation (1), for instance, the contrastive loss for each sample i is computed by considering all positive samples $p\in P\left(i\right)$, where $P\left(i\right)$ represents the set of positive samples for the anchor i. The SupCon loss then evaluates the distance between the anchor and all positive samples relative to a set of negatives (samples that belong to different classes). The function inside the log term measures the similarity between the anchor $z_{i}$ and a positive sample $z_{p}$, normalized by the sum of exponentials of similarities between the anchor and all other samples $z_{a}\in A\left(i\right)$, where $A\left(i\right)$ is the set of all samples except the anchor. This is scaled by a temperature parameter τ to control the sharpness of the distribution. All our experiments used a temperature of τ = 0.07. Lower temperatures benefit training more than higher ones, but extremely low temperatures are harder to train due to numerical instability [40,51,52]. This formulation enables the incorporation of multiple positive samples, making it particularly well suited for supervised learning environments [50]. The contrastive loss is defined as follows:

(1) $$L_{contrasive}=\sum_{i\in I}\frac{-1}{\left|P\left(i\right)\right|}\sum_{p\in P\left(i\right)}\log\frac{\exp\left(\frac{\mathrm{sim}\left(z_{i},z_{p}\right)}{\tau }\right)}{\sum_{a\in A\left(i\right)}\exp\left(\frac{\mathrm{sim}\left(z_{i},z_{a}\right)}{\tau }\right)}$$

where

$z\in R^{N\times d}$ represents the normalized projections of the input samples into an embedding space, where N is the number of samples and d is the dimensionality of the embedding.

*i* ∈ *I* refers to each sample in the batch.

*P*(*i*) is the set of positive samples for the anchor *i*.

*A*(*i*) is the set of all other samples excluding the anchor.

τ is a temperature parameter to scale the similarities, typically set at τ=0.07.

$\mathrm{sim}\left(z_{i},z_{p}\right)$ denotes the cosine similarity between the projections $z_{i}$ and $z_{p}$.

SupCon fundamentally differs from traditional classification by optimizing the geometric structure of the learned representation space [40]. While cross-entropy loss only ensures correct class prediction, SupCon explicitly ensures that samples from the same class have similar representations while pushing apart representations from different classes [40]. For PD detection, this approach can be particularly beneficial as it encourages more robust representations.

In datasets with numerous easy-to-contrast pairs, contrastive loss gradients can become minimal, hindering informative updates [50,53]. To mitigate this, we employ hard pair scaling with a factor of 1.5, identifying the most challenging negative examples through cosine similarity analysis. It should be noted that, while SupCon loss aims to distinguish pairs across classes, we complement this with a SoftMax-based cross-entropy loss [40], blocking its gradient to prevent encoder updates during training.

### 2.5. Baseline Features and Models

A baseline approach was established using traditional acoustic feature extraction. Two primary feature sets were selected, MFCCs and eGeMAPS, known for their ability to distinguish pathological from healthy speech [23,32,54,55]. MFCCs capture the spectral envelope characteristics of speech by modeling the human auditory system’s frequency perception, making them particularly sensitive to the vocal tract changes and phonatory alterations commonly observed in PD speech [37]. MFCCs were computed using Hamming-windowed frames of 25 ms with a 5 ms shift, producing a 39-dimensional vector per utterance, including cepstral coefficients and their derivatives, via the Librosa library [56]. eGeMAPS (the extended Geneva minimalistic acoustic parameter set) [42], designed to capture key vocal characteristics, comprised 88 features, including prosodic, excitation, vocal tract, and spectral descriptors, extracted using the openSMILE toolkit (2.5.0) [32,57]. (A detailed list of these 88 features and their associated functionals is available in Supplementary Table S3). The combination of temporal, spectral, and prosodic features in eGeMAPS makes it particularly well suited for detecting the multi-dimensional speech impairments associated with PD, including changes in fundamental frequency, jitter, shimmer, and harmonic-to-noise ratios [37].

The extracted features were classified using an XGBoost model (2.1.2) [41] and a multilayer perceptron (MLP) with a predefined architecture. For XGBoost, we conducted a thorough hyperparameter search and selected the best-performing parameters, as detailed in the Supplementary Materials. For the MLP model, we used the same classification head architecture as in the ASR models to ensure consistency. These classifiers, utilizing these well-established acoustic features, predicted speech categories and served as a robust baseline for comparison with the proposed ASR-based deep learning methods.

### 2.6. Experimental Setup and Training Parameters

We utilized several Python (3.10.0) libraries in our implementation, including PyTorch (2.3.0) [46] for developing deep learning models, Pandas (2.2.2) [58] and NumPy (1.24.3) [59] for data manipulation and analysis, and Matplotlib (3.4.3) [60], along with Scikit-learn (1.3.0) [61], for visualization and certain analytical tasks.

As detailed in Table 1, key training hyperparameters used during model optimization included the learning rate, batch size, and number of epochs. The models were trained using an Adaptive Moment Estimation optimizer with Weight Decay (AdamW), an optimization algorithm with cross-entropy loss, to measure prediction error [62]. A learning rate of 2 × 10^−5^ was set initially and adjusted over time using a scheduler. We carried out the experiments using a system comprising an Intel Core i7 13700K CPU @ 5.2 GHz, with 128GB of RAM and GPU NVIDIA RTX 4090 24GB.

### 2.7. Evaluation Metrics

This study employed five primary evaluation criteria: the accuracy (AC), F1 score, specificity (SP), sensitivity (SN), and precision (P). The equations for these metrics are presented in Equations (2)–(6).

(2) $$Accuracy=\frac{TP+TN}{TP+TN+FN+\;FP}$$

(3) $$\begin{array}{c} Sensitivity=\frac{TP}{TP+FN} \end{array}$$

(4) $$Specificity=\frac{TN}{TN+FP}$$

(5) $$\begin{array}{c} Precison=\frac{TP}{TP+FP} \end{array}$$

(6) $$\begin{array}{c} F1=\frac{2\times Recall\times Precison}{Precison+Recall} \end{array}$$

where *TP*, *TN*, *FP*, and *FN* represent the counts of true positives, true negatives, false positives, and false negatives, respectively. The sensitivity and specificity are important statistical metrics that indicate the proportion of correctly identified positive and negative cases.

### 2.8. The Grad-CAM Technique for Visual Explanations

Grad-CAM (Gradient-weighted Class Activation Mapping) is a technique that enables the visualization of which regions of an input—such as an audio waveform or spectrogram—contribute most to a model’s prediction [43]. It can be adapted for ASR transformer models to visualize prediction relevance in audio data. By overlaying Grad-CAM heatmaps on audio waveforms, researchers can identify which time segments contain critical features for model inference [63]. The technique involves computing gradients of the target output with respect to attention weights in the final transformer layer, allowing the visualization of the most contributory audio signal parts for tasks like Parkinson’s disease classification. A general architecture illustrating the Grad-CAM mechanism applied in this context is provided in Supplementary Figure S2.

## 3. Results and Discussion

This section presents a comprehensive analysis of the results, prominently demonstrates the superior performance of ASR-based features enhanced by SupCon Learning over traditional methods for PD detection and assesses the performance of all models examined in this study.

### 3.1. Classification Performance

**Figure 3:**
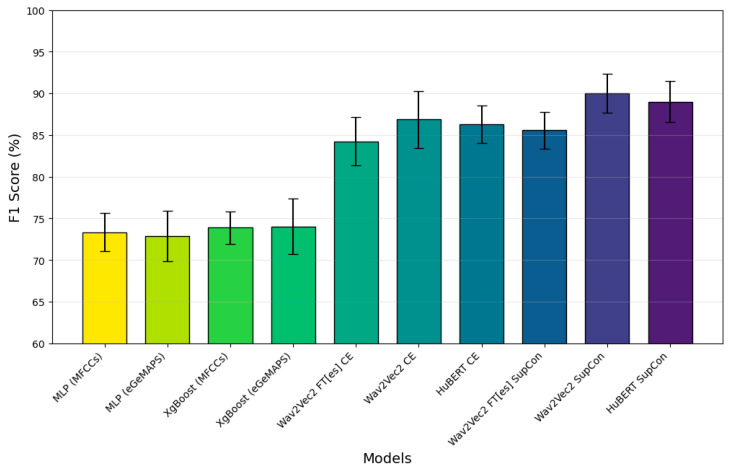
F1 score (%) comparison across different models, showing the superior performance of SupCon learning approaches over CE-based models. Wav2Vec 2.0 SupCon and HuBERT SupCon achieve the highest F1 scores of approximately 90%, with error bars indicating model performance variability. In order to enable a clear comparison, the accuracy scale starts at 60%.

To assess the models’ performance in a fair and comprehensive manner, we divided the dataset into two parts, 80% for training and 20% for testing, ensuring that there was no speaker overlap between them. The test set served as the final evaluation benchmark. Using the training set, a stratified speaker-independent 5-fold cross-validation approach was implemented for all experiments. The data was divided into five folds, ensuring that there was no overlap of patients between folds, to prevent data leakage. The model was trained on four folds (80% of training data) and assessed on the remaining fold (20%), with this process repeated five times so that each fold served as the evaluation set once. Early stopping was employed during training to prevent overfitting by saving the best-performing model based on validation performance. Finally, we evaluated the best model’s generalization capability on the previously held-out test set. This method provided a rigorous assessment of model performance on unseen data. The performance of each model on the test set, including the precision, recall, F1 score, and accuracy, is detailed in Table 2. The graph representation of the corresponding performance (F1 score) for each model is shown in Figure 3.

Across all models, training with SupCon consistently yields better F1 scores, precision, and sensitivity compared to CE alone. Specifically, Wav2Vec 2.0 with SupCon achieves the highest F1 score of 90.0%, along with improved performance for other metrics, indicating that SupCon training improves the model’s ability to accurately identify and classify relevant patterns.

The bar graph displays the F1 scores of these models, with Wav2Vec 2.0-base and HuBERT with SupCon emerging as the top-performing configurations. HuBERT’s performance closely matched that of Wav2Vec 2.0. The baseline models (XGBoost and MLP) trained on acoustic features achieved F1 scores between 73.33% and 74.52% and AUC scores between 0.80 and 0.81. These results were significantly lower than those of our ASR-based models, demonstrating the superior performance of fine-tuned ASR models in PD detection. This highlights a key advantage of our approach, as transfer learning further boosts performance by leveraging pre-trained models from related speech tasks, enabling the adaptation of rich representations learned using large general datasets for more specialized tasks like medical diagnosis, particularly when the target-domain data is scarce.

Among the models, MLP (MFCC features) and XGBoost (MFCC features) exhibit the lowest F1 scores. Of these, XGBoost and MLP training with egeMAPS features perform slightly better, but both are outperformed by the other models.

The error bars demonstrate the variability across runs, with the highest-performing models exhibiting the lower variability. These results emphasize the effectiveness of SupCon training across both ASR models, while fine-tuning for Spanish (Wav2Vec 2.0-FT[es]) shows a competitive yet slightly lower performance, suggesting some limitations in the transferability or adaptation of the model across languages.

**Figure 4:**
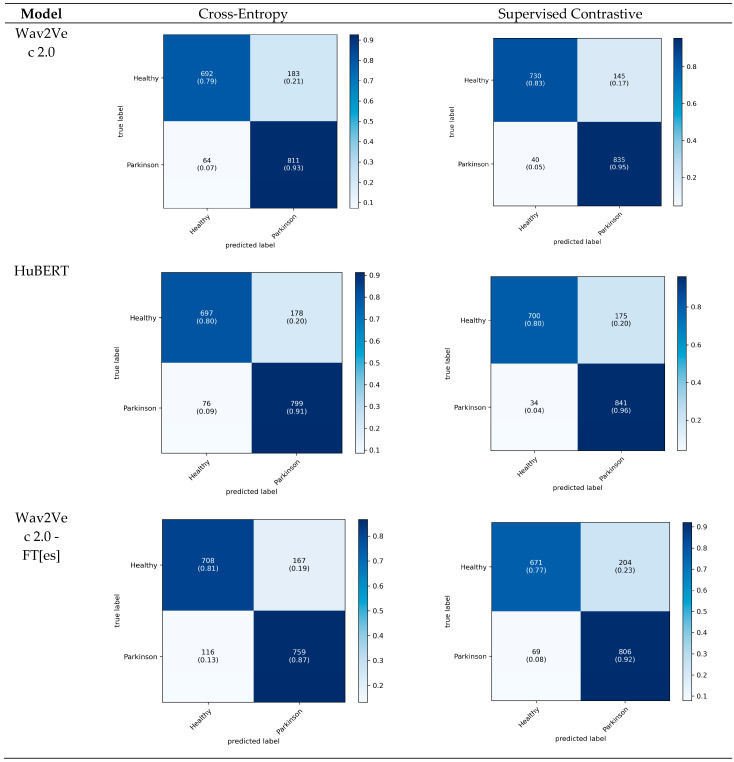
Cumulative confusion matrices depicting each model’s performance across five cross-validation folds on the sentence dataset.

The proposed models were also assessed via cumulative confusion matrices and receiver operating characteristic (ROC) curves across 5-fold cross-validation. The cumulative confusion matrices provide a comprehensive view of model performance by summarizing the results across all folds. The ROC curves illustrate the tradeoff between the true positive rate and the false positive rate, offering insights into the diagnostic power of the models. The area under the ROC curve (AUC) serves as an indicator of model performance, with higher AUC values reflecting a superior classification ability. The cumulative confusion matrices in Figure 4 allow us to evaluate the diagnostic performance of three models trained with CE and SupCon across five cross-validation folds.

In the context of diagnosing PD, recall (sensitivity) is particularly critical because Parkinson’s is a progressive neurodegenerative disorder in which early detection is essential in managing symptoms and potentially slowing disease progression. A high recall ensures that the model captures as many true Parkinson’s cases as possible, minimizing false negatives that could delay essential treatment. Among the models, Wav2Vec 2.0 demonstrates the most balanced performance, achieving a high sensitivity (92.6%) while maintaining competitive accuracy across both classes. HuBERT follows closely but shows a slightly lower sensitivity for Parkinson’s cases (91.3%), whereas the Wav2Vec 2.0-FT[es] model, although effective, has a lower recall for healthy cases (73.8%) and performs best in identifying Parkinson’s cases (87.7%). This analysis shows that while all three models are good at differentiating between the healthy and Parkinson’s classes, Wav2Vec 2.0’s superior recall makes it particularly suitable for the early diagnosis of PD, where maximizing sensitivity is essential in improving patient outcomes through timely treatment. Additionally, the cumulative confusion matrices for the baseline models are provided in Supplementary Figure S3.

**Figure 5:**
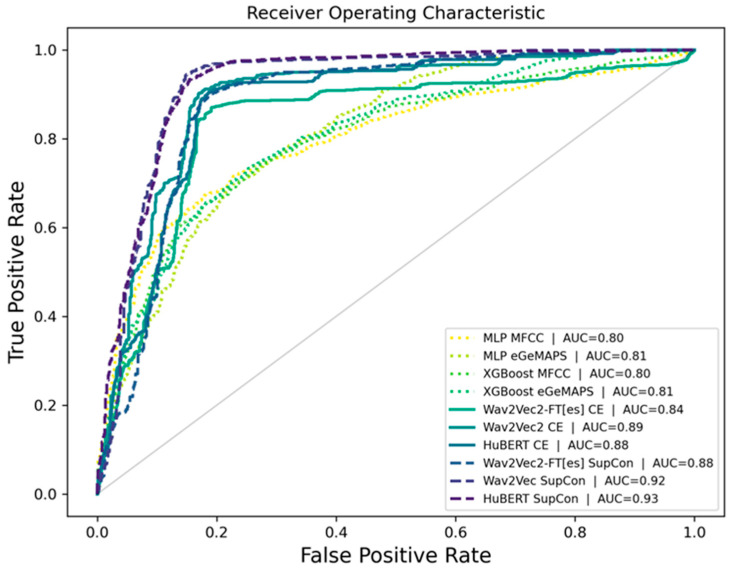
ROC curves comparing the traditional model with the Wav2Vec 2.0 and HuBERT model variants, with SupCon approaches achieving the highest performance (AUC = 0.92–0.93) compared to cross-entropy (CE)-based models (AUC = 0.84–0.89).

Figure 5 presents the ROC curves comparing the performance of different model variants and training approaches. Overall, the baseline models (XGBoost and MLP) demonstrated lower performance (AUC < 0.81) compared to the ASR-based models. The SupCon methods demonstrated superior performance, with HuBERT SupCon and Wav2Vec SupCon achieving the highest AUC scores of 0.93 and 0.92, respectively, placing these results competitively within the 0.77–0.98 AUC range previously reported for Wav2Vec 2.0 in cross-database PD classification studies. These results significantly outperformed the cross-entropy-based approaches, which showed comparatively lower performance with AUC values ranging from 0.84 to 0.89. Specifically, Wav2Vec 2.0-FT[es] CE showed the lowest performance with an AUC of 0.84, while Wav2Vec 2.0 CE and HuBERT CE performed moderately better with AUC scores of 0.89 and 0.88, respectively. The steeper curves and higher true positive rates at lower false positive rates for the SupCon models indicate their enhanced ability to discriminate between classes compared to their CE-based counterparts.

Furthermore, we analyzed the classification accuracy for each of the 16 sentence types to identify utterances that were consistently more or less challenging for the models (Figure S4). Each sentence is associated with a specific speech assessment task—such as velopharyngeal closure, prosody, or intonation-emotion—and is labeled with a unique ID (e.g., ABLANDADA, PIDIO, BURRO) (Tables S1 and S2). The analysis showed clear variation in performance across sentence types. Sentences like PERRO, ACAMPADA, and ABLANDADA, which primarily focus on simpler articulatory features or velopharyngeal closure, achieved the highest average accuracies (≥0.89). In contrast, sentences such as CALLE, MANGA, and VINO, which include more nuanced prosodic or emotional intonation components, recorded the lowest average accuracies (≤0.85). This pattern suggests that while ASR-based models are generally effective, their performance may be hindered by increased prosodic complexity and emotional expressiveness, potentially impacting their ability to robustly capture Parkinson’s disease-related speech biomarkers.

In terms of model performance, Wav2Vec 2.0 consistently outperformed HuBERT, particularly when combined with SupCon loss, achieving accuracies between 0.87 and 0.94, compared to 0.83–0.89 with CE loss. The largest gain is observed for the “ABLANDADA” sentence, for which the accuracy reaches 0.94, showcasing this combination of model and loss function as particularly effective. The HuBERT model performs slightly less well but still effectively, with accuracies of 0.84–0.91 using SupCon loss and 0.83–0.89 with CE. Overall, SupCon loss consistently outperforms CE across both models, with Wav2Vec 2.0 emerging as the most effective model for PD detection using this speech dataset.

To contextualize our results within the current research landscape, we compared our findings with recent methods that employ similar ASR-based approaches for PD detection. Table 3 presents a comparison of our best-performing model (Wav2Vec 2.0 + SupCon) with key benchmarks from the literature. Javanmardi et al. achieved 85–92% accuracy using pre-trained models for dysarthria detection [32]. Klempíř et al. reported 0.77–0.98 AUROC for Wav2Vec embeddings in cross-database PD classification [36]. Chronowski et al. achieved 97.92% accuracy using Wav2Vec 2.0 on smartphone-collected speech [39]. While the datasets used across these studies differ—limiting direct comparability—our approach achieved a 90.0% F1 score and 0.92 AUC, positioning it competitively within these ranges while introducing the integration of supervised contrastive learning. Notably, our integration of SupCon learning yielded consistent performance improvements (F1 score: 90.0% vs. 86.86%), highlighting its potential in enhancing existing ASR-based PD detection systems.

It is important to note that while our study demonstrates promising results for ASR-based models using Castilian speech data with a relatively small sample size, the single-language nature of the dataset represents a limitation with regard to broader applicability. Cross-linguistic research has shown that speech-based PD detection models can exhibit varying performance across different languages due to distinct phonetic structures, prosodic patterns, and linguistic characteristics [37,64,65]. For instance, features learned by ASR models pre-trained or fine-tuned on Spanish speech might not optimally capture the acoustic manifestations of PD in languages with substantially different phonological systems or prosody. This context inherently limits the generalizability of our findings to other linguistic settings and broader populations and may require adaptation or retraining for other languages.

### 3.2. Grad-CAM Feature Visualization

**Figure 6:**
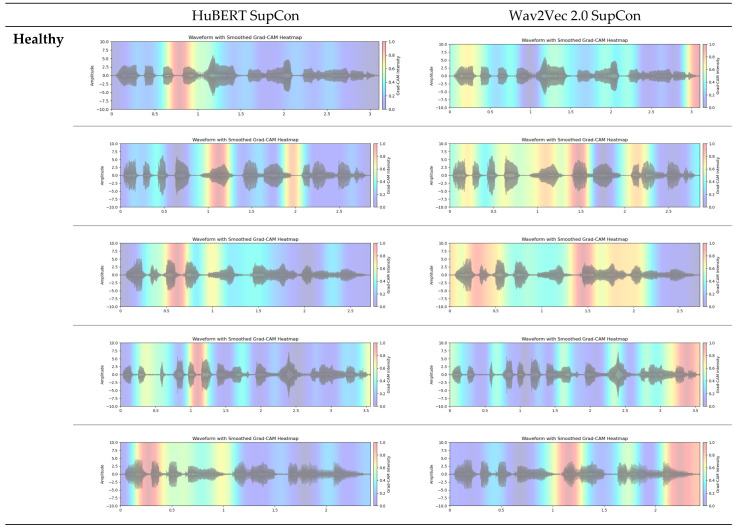

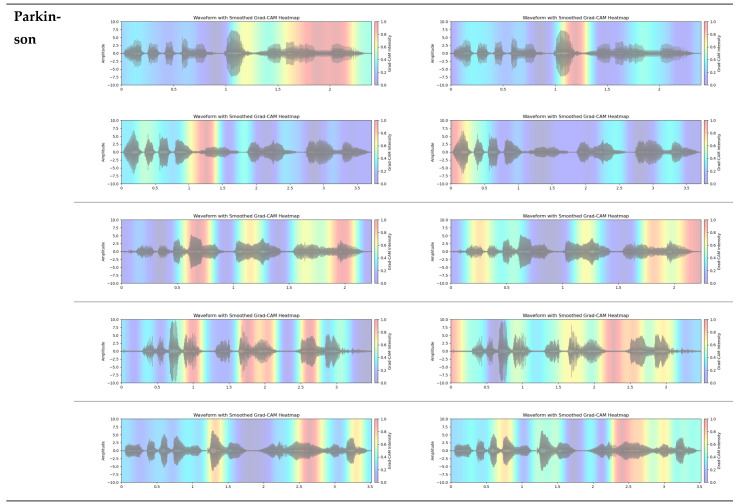
Grad-CAM visualization features of models for different classes.

Figure 6 shows Grad-CAM feature map visualizations for 10 sample audio clips taken from our dataset (ABLANDADA sentence). To ensure a meaningful comparison between Grad-CAM and t-SNE visualizations, we exclusively used models from the final fold. Grad-CAM analysis reveals which regions of the input contribute most significantly to the model’s predictions. For this visualization, we focused on the output projections from the final self-attention layer. This layer encapsulates the highest-level features processed by the model. It directly influences the model’s output by projecting the attention mechanism’s output back into the embedding space, making it a good candidate for identifying the input regions that contribute most significantly to the final decision.

Both models highlight regions within the waveforms, but with differing intensity and spread, which suggests that they may be emphasizing slightly different acoustic features for classification. Regarding the healthy class, the wav2vec model’s Grad-CAM heatmaps generally exhibit more consistent and expansive high-activation zones across the waveform compared to HuBERT, potentially indicating a broader approach to feature extraction. Conversely, HuBERT shows narrower, more localized regions of attention, suggesting that it might be focusing on more specific, fine-grained features of the audio signal. When analyzing Parkinson’s speech samples, both models demonstrate a consistent focus on the central regions of the audio waveforms. In most cases, their focus is quite similar. A notable observation is that the HuBERT model appears to strongly activate voiced segments in Parkinson’s speech while paying attention to small unvoiced sections in healthy speech samples. This variation in activation regions could reflect differences in how each model captures the temporal structure of speech that are relevant in distinguishing healthy speech patterns from Parkinsonian ones.

While these visualizations may broadly align with known PD speech characteristics (e.g., phonatory or rhythmic changes), a significant limitation of this study is the lack of a detailed correlation of these highlighted regions with specific clinical speech impairments. Such an in-depth analysis, which would require direct collaboration with speech pathology experts for fine-grained interpretation, was not conducted but remains critical for future research to enhance clinical interpretability. Nevertheless, even at this stage, the application of Grad-CAM can provide clinicians with user-friendly visualizations, highlighting the audio regions that influence a model’s detection of PD. This capability allows clinicians to align the model’s decisions with their medical knowledge, thereby increasing confidence in the model’s reliability and identifying areas for improvement if the heatmap shows activation in irrelevant audio sections.

In addition, to assess the impact of different feature extraction methods and training objectives on learned representations, we visualized feature embeddings using t-SNE. Figure S5 compares the feature space of traditional acoustic features (MFCC, eGeMAPS) with embeddings from ASR models trained using cross-entropy and SupCon loss. The t-SNE visualizations reveal that traditional acoustic features (MFCC and eGeMAPS) show poor clustering patterns with significant overlap between PD and healthy samples, indicating limited discriminative power for complex PD speech patterns. In contrast, ASR-based models show superior feature space organization. Cross-entropy- and SupCon-trained models both exhibit well-separated clusters, with SupCon-trained models demonstrating slightly more compact and better-separated clusters compared to CE models. However, the improvement from CE to SupCon, while consistent, is more subtle than the significant difference between traditional features and ASR-based approaches.

### 3.3. Ablation Study

In this section, we present the results of several ablation experiments to evaluate the impact of different components in our proposed integrated SupCon training. We specifically examine how factors such as the projection size, temperature settings, freezing the encoder, utilizing features from various layers, and the impact of not scaling hard negatives influence performance.

Table 4 shows the ablation results for Wav2Vec 2.0 and HuBERT with SupCon across different projection head sizes. For Wav2Vec 2.0, the best accuracy (89.43%) and F1 score (90.00%) were achieved with a projection head size of 32. However, larger head sizes result in a slight decline in performance, with the accuracy dropping to 87.43% for a head size of 256. Similarly, for HuBERT, the highest performance is observed with a projection head size of 32, achieving an accuracy of 88.06% and F1 score of 88.99%. Increasing the projection head size also leads to a marginal decrease in performance for HuBERT, with the accuracy falling to 87.60% for a head size of 256.

Table 5 presents the performance metrics of three models, Wav2Vec 2.0 + CE, HuBERT+ CE, and Wav2Vec 2.0-FT[es] + CE, with and without fine-tuning. Fine-tuning consistently improved the performance across all models. Notably, HuBERT+ CE with fine-tuning achieved the highest accuracy (85.49%) and sensitivity (91.31%), indicating robust recognition capabilities. In contrast, Wav2Vec 2.0-FT[es] + CE, despite showing significant improvements post fine-tuning, had the lowest scores without fine-tuning, suggesting a dependency on additional training for optimal performance. The results underscore the importance of fine-tuning in enhancing model effectiveness, particularly for achieving higher specificity and sensitivity.

Wav2Vec 2.0 and HuBERT, both coupled with SupCon, were evaluated regarding their classification performance at different temperatures (0.03, 0.07, and 0.10), as detailed in Table 6. Wav2Vec 2.0 achieved its highest accuracy (89.43% ± 2.31%) and F1 score (90.00% ± 2.35%) at a temperature of 0.07. HuBERT with SupCon showed comparable performance at 0.03 and 0.07 but a significant drop at 0.10, despite higher sensitivity. The optimal temperature appears model-specific, with 0.07 favoring Wav2Vec and HuBERT.

We also conducted an ablation study on feature extractor layers (Table S4) and the impact of scaling hard negatives with different projection sizes in SupCon (Table S5). For CE loss, both Wav2Vec 2.0 and HuBERT showed improved performance with deeper layers, peaking at Layer 12. Wav2Vec 2.0 achieved 85.89% accuracy (±4.27) and 86.86% F1 score (±3.42), while HuBERT reached 85.49% accuracy (±2.41) and 86.27% F1 score (±2.26). Sensitivity peaked at Layer 12 for both models, though specificity showed higher variability. For SupCon criteria, scaling hard negatives consistently improved metrics, with Wav2Vec 2.0 outperforming HuBERT, particularly in terms of the accuracy, F1 score, and precision. A projection size of 32 outperformed 64 for both models, with metrics showing stable variations (2–4%). Detailed results are provided in Tables S4 and S5 in the Supplementary Materials.

## 4. Conclusions

This study highlights the potential of leveraging ASR models for the detection and monitoring of PD through speech analysis utilizing the NeuroVoz voice and speech database. In these experiments, classification systems were developed using two popular pre-trained ASR models, Wav2Vec 2.0 and HuBERT, for feature extraction, alongside traditional baseline features and the base models (XGBoost and MLP). The ASR-based models were trained using two approaches: straightforward cross-entropy and SupCon learning, aimed at accurately predicting the output labels. The results showed that features from pre-trained models outperformed the baseline features, suggesting that unsupervised training on diverse healthy speech data provides a strong foundation for pathological voice detection.

Our approach yielded promising results, with Wav2Vec 2.0 achieving an F1 score of up to 90.00% in distinguishing PD from healthy individuals. Furthermore, the application of SupCon enhanced the model’s performance, improving the ability to discern subtle speech changes associated with PD. In this context, the contrastive learning strategy improved Wav2Vec 2.0’s performance from 0.89 and 86.86% to 0.92 and 90.00% for the metrics of the AUROC and F1 score. The integration of gradient-based feature importance methods, such as Grad-CAM, allowed us to visualize and understand the specific regions of the speech signal that contributed most to the model’s decisions, adding a layer of interpretability.

In conclusion, these results underscore the efficacy of ASR models along with contrastive learning in non-invasive PD diagnosis and monitoring, paving the way for their potential application in clinical settings. This offers a non-invasive approach to early detection and ongoing management, addressing the critical need for objective and accessible tools in healthcare. However, it is crucial that we acknowledge the limitations of this study, including the use of a single-language dataset and a relatively small cohort. These factors may restrict the direct generalizability of our findings. The impact of linguistic variability on model performance warrants careful consideration and further dedicated research. Future research will aim to extend this method to cross-linguistic PD prediction, thereby contributing to the development of more robust and universally applicable diagnostic tools.

## Figures and Tables

**Table 1:** Parameter settings for training models.

Parameter	Values
Epochs	100
Batch size	32
Initial learning rate	2 × 10^−5^
Optimizer	AdamW (β_1_ = 0.9, β_2_ = 0.999, Weight decay = 0.01)
Loss function	Cross-entropy loss and supervised contrastive loss
Dropout	0.2

**Table 2:** Summary of the classification performance on the test set (mean ± sample standard deviation) for each model, comparing the precision, recall, F1 score, and accuracy across the different models.

	Metric	AC [%]	F1 [%]	P [%]	SN [%]	SP [%]
Model						
MLP						
+MFCC Features		72.51 (±2.28)	73.33 (±2.85)	71.14 (±1.82)	75.77 (±4.99)	69.26 (±2.84)
+eGeMAPS Features		72.86 (±3.05)	74.51 (±3.03)	70.19 (±2.47)	79.43 (±3.98)	66.29 (±2.77)
XgBoost						
+MFCC Features		72.74 (±2.02)	73.86 (±1.02)	70.98 (±2.10)	77.03 (±2.60)	68.46 (±2.95)
+eGeMAPS Features		73.60 (±3.19)	74.02 (±3.36)	72.83 (±2.96)	75.31 (±4.49)	71.89 (±3.60)
Wav2Vec 2.0						
+CE		85.89 (±4.27)	86.86 (±3.42)	82.02 (±5.89)	92.69 (±4.30)	79.09 (±9.09)
+ SupCon		89.43 (±2.31)	90.00 (±2.35)	85.31 (±2.77)	95.43 (±4.83)	83.43 (±4.00)
HuBERT						
+CE		85.49 (±2.41)	86.27 (±2.26)	82.10 (±24)	91.31 (±5.86)	79.66 (±6.88)
+ SupCon		88.06 (±2.97)	88.99 (±2.48)	83.02 (±24)	96.11 (±3.11)	80.00 (±6.60)
Wav2Vec 2.0-FT [Spanish]						
+CE		83.83 (±2.45)	84.21 (±2.90)	82.24 (±3.63)	86.74 (±7.33)	80.91 (±6.14)
+ SupCon		84.40 (±2.84)	85.56 (±2.18)	80.17 (±4.83)	92.11 (±4.61)	76.69 (±8.18)

**Table 3:** Comparison with recent ASR-based PD detection methods.

Study	Method	Dataset	Performance
Javanmardi et al. [32]	Wav2Vec 2.0 and HuBERT	UA-Speech	85–92% accuracy
Klempíř et al. [36]	Wav2Vec embeddings	Multiple	0.77–0.98 AUC
Chronowski et al. [39]	Wav2Vec 2.0	Smartphone data	97.92% accuracy
Our study	Wav2Vec 2.0 + SupCon	NeuroVoz	90.0% F1, 0.92 AUC

**Table 4:** Classification performance of Wav2Vec 2.0 and HuBERT with SupCon across different projection head sizes, showing accuracy, precision, recall, F1 score, and AUC.

	Metric	Projection Head Size	AC [%]	F1 [%]	P [%]	SN [%]	SP [%]
Model							
Wav2Vec 2.0 + SupCon							
		32	89.43 (±2.31)	90.00 (±2.35)	85.31 (±2.77)	95.43 (±4.83)	83.43 (±4.00)
		64	89.09 (±1.75)	89.63 (±1.53)	85.65 (±3.47)	94.17 (±3.29)	84.00 (±4.83)
		128	87.60 (±1.33)	88.17 (±1.42)	84.25 (±1.67)	92.57 (±3.45)	82.63 (±2.41)
		256	87.43 (±2.99)	88.29 (±2.49)	83.01 (±3.93)	94.40 (±2.15)	80.46 (±5.79)
HuBERT+ SupCon							
		32	88.06 (±2.97)	88.99 (±2.48)	83.02 (±4.52)	96.11 (±3.11)	80.00 (±6.60)
		64	87.83 (±1.55)	88.72 (±1.38)	82.77 (±2.27)	95.66 (±2.51)	80.00 (±3.38)
		128	87.49 (±2.57)	88.45 (±2.03)	82.67 (±4.73)	95.43 (±3.90)	79.54 (±7.24)
		256	87.60 (±0.87)	88.42 (±0.65)	83.07 (±2.40)	94.63 (±2.67)	80.57 (±3.77)

**Table 5:** Performance metrics for three speech recognition models (Wav2Vec 2.0, HuBERT, and Wav2Vec 2.0-FT[es]) with (√) and without fine-tuning (**×**).

	Metric	Fine-Tuning of Model?	AC [%]	F1 [%]	P [%]	SN [%]	SP [%]
Model							
Wav2Vec 2.0 + CE							
		√	85.89 (±4.27)	86.86 (±3.42)	82.02 (±5.89)	92.69 (±4.30)	79.09 (±1.97)
		×	70.29 (±1.82)	65.36 (±5.35)	78.39 (±4.72)	57.14 (±10.55)	83.43 (±7.45)
HuBERT+ CE							
		√	85.49 (±2.41)	86.27 (±2.26)	82.10 (±24)	91.31 (±5.86)	79.66 (±6.88)
		×	82.69 (±1.21)	84.30 (±0.96)	77.17 (±1.68)	92.91 (±1.32)	72.46 (±2.69)
Wav2Vec 2.0-FT[es] + CE							
		√	83.83 (±2.45)	84.21 (±2.90)	82.24 (±3.63)	86.74 (±7.33)	80.91 (±6.14)
		×	65.54 (±2.05)	84.21 (±62.95)	67.97 (±1.73)	58.74 (±4.94)	72.34 (±3.34)

**Table 6:** Classification performance of Wav2Vec 2.0 and HuBERT with SupCon at different temperatures, showing accuracy, precision, recall, F1, AUC, and specificity with standard deviations.

	Metric	Temperature	AC [%]	F1 [%]	P [%]	SN [%]	SP [%]
Model							
Wav2Vec 2.0 + SupCon							
		0.03	87.43 (±1.34)	87.86 (±1.24)	85.11 (±5.09)	90.97 (±3.49)	79.66 (±7.34)
		0.07	89.43 (±2.31)	90.00 (±2.35)	85.31 (±2.77)	95.43 (±4.83)	83.43 (±4.00)
		0.10	87.89 (±2.82)	88.32 (±2.67)	85.39 (±3.73)	91.66 (±4.6)	84.11 (±5.12)
HuBERT + SupCon							
		0.03	87.54 (±3.58)	88.52 (±3.03)	82.70 (±5.09)	95.43 (±2.91)	79.66 (±7.34)
		0.07	88.06 (±2.97)	88.99 (±2.48)	83.02 (±4.52)	96.11 (±3.11)	80.00 (±6.60)
		0.10	86.69 (±0.87)	87.97 (±1.57)	80.55 (±3.45)	97.03 (±1.48)	76.34 (±5.40)

## Data Availability

The dataset used in this study is publicly available upon submitting a request. For access to the NeuroVoz dataset, please visit: https://zenodo.org/records/10777657. The pre-trained models utilized in this study include ‘facebook/wav2vec2-base-10k-voxpopuli-ft-en’, ‘facebook/hubert-base-ls960’, and ‘facebook/wav2vec2-base’, all of which are available in the Hugging Face model repository. The source code is also available on request.

## Supplementary Materials

The following supporting information can be downloaded at: https://www.mdpi.com/article/10.3390/bioengineering12070728/s1, Table S1. Transcriptions in the IPA and translations of selected sentences; Table S2. Overview of speech assessment tasks focusing on velopharyngeal closure, articulation, prosody, intonation, and other vocal functions; Section S1: eXtreme Gradient Boosting (XGBoost); Figure S1. Histogram showing the distribution of audio lengths for the tasks across the groups: HC and PD; Figure S2. Grad-CAM architecture employed for visual explanation.; Figure S3. The cumulative confusion matrices show the performance of each model across fivefold cross-validation on the dataset of sentences; Figure S4. Accuracy comparison across various sentences; Figure S5. t-SNE visualizations of feature representations for Parkinson’s detection; Table S3. Overview of acoustic features and their associated functionals in eGeMAPS, totaling 88 features; Table S4. Classification performance of Wav2Vec 2.0 and HuBERT with cross-entropy using different layers as feature extractors, showing accuracy, precision, recall, F1, AUC, and specificity with standard deviations; Table S5. Classification performance of Wav2Vec 2.0 and HuBERT with SupCon with and without scaling hard negatives and across two different projection sizes (32 and 64), showing accuracy, precision, recall, F1, AUC, and specificity with standard deviations. References [19,41,44,66] are cited in supplementary materials.

## Abbreviations

The following abbreviations are used in this manuscript:

AUC	Area Under the Receiver Operating Characteristic Curve
ASR	Automatic Speech Recognition
CE	Cross-Entropy
CNN	Convolutional Neural Network
DL	Deep Learning
DDK	Diadochokinetic
eGeMAPS	extended Geneva Minimalistic Acoustic Parameter Set
FN	False Negative
FP	False Positive
FT	Fine-Tuned
F1	F1 Score (harmonic mean of precision and recall)
Grad-CAM	Gradient-weighted Class Activation Mapping
HC	Healthy Control
kNNs	k-Nearest Neighbors
MLP	Multilayer Perceptron
MFCCs	Mel-Frequency Cepstral Coefficients
ML	Machine Learning
PD	Parkinson’s Disease
RNN	Recurrent Neural Network
ROC	Receiver Operating Characteristic
SVM	Support Vector Machine
SN	Sensitivity (Recall)
SP	Specificity
SupCon	Supervised Contrastive Learning
TN	True Negative
TP	True Positive
UPDRS	Unified Parkinson’s Disease Rating Scale
Wav2Vec 2.0	A self-supervised speech model developed by Facebook AI
τ	Temperature parameter (used in contrastive loss function)
