# Supplementary material for: Speech-Based Parkinson’s Detection Using Pre-Trained Self-Supervised Automatic Speech Recognition (ASR) Models and Supervised Contrastive Learning
Source: Bioengineering (Basel). 2025 Jul 1;12(7):728. doi: 10.3390/bioengineering12070728 (PMC12292552; doi:10.3390/bioengineering12070728)
Supplement: Supplementary file 1 [file bioengineering-12-00728-s001.zip › bioengineering-3684287-supplementary.pdf]

Speech-Based Parkinson's Detection Using Pre-Trained Self-Supervised ASR Models and Supervised Contrastive Learning

Hadi Sedigh Malekroodi [1], Nuwan Madusanka [2], Byeong-il Lee [1,2,3] and Myunggi Yi [1,2,4,*]

[1] Industry 4.0 Convergence Bionics Engineering, Pukyong National University, Busan 48513, Republic of Korea; hadi_sedigh@pukyong.ac.kr (H.S.M.); bilee@pknu.ac.kr (B.-i.L.)
[2] Digital Healthcare Research Center, Institute of Information Technology and Convergence, Pukyong National University, Busan 48513, Republic of Korea; nuwanmadusanka@hotmail.com
[3] Major of Human Bioconvergence, Division of Smart Healthcare, Pukyong National University,
Busan 48513, Republic of Korea
[4] Major of Biomedical Engineering, Division of Smart Healthcare, Pukyong National University,
Busan 48513, Republic of Korea
* Correspondence: myunggi@pknu.ac.kr

ORCID iD:

Hadi Sedigh Malekroodi: 0009-0001-1958-1525
Nuwan Madusanka: 0000-0001-7982-1036
Byeong-il Lee: 0000-0002-1574-7145
Myunggi Yi: 0000-0003-4864-959X

Supplementary materials include:

Table S1. Transcriptions in the IPA and translations of selected sentences

Table S2. Overview of speech assessment tasks focusing on Velopharyngeal Closure , articulation, prosody, intonation, and other vocal functions

Section S1: eXtreme Gradient Boosting (XGBoost)

Figure S1. Histogram showing the distribution of audio lengths for the tasks across the groups: HC and PD.

Figure S2. Grad-CAM architecture employed for visual explanation.

Figure S3. The cumulative confusion matrices show the performance of each model across five folds of cross-validation on the dataset of sentences.

Figure S4. Accuracy comparison across various sentences.

Figure S5. t-SNE visualizations of feature representations for Parkinson's Detection.

Table S3. Overview of acoustic features and their associated functionals in eGeMAPS, totaling 88 features

Table S4. Classification performance of Wav2Vec 2.0 and HuBERT with Cross Entropy using different layer as feature extractor, showing accuracy, precision, recall, F1, AUC, and specificity with standard deviations.

Table S5. Classification performance of Wav2Vec 2.0 and HuBERT with SupCon at with and without scaling hard negatives, and across two different projection sizes (32 and 64), showing accuracy, precision, recall, F1, AUC, and specificity with standard deviations.

Table S1. Transcriptions in the IPA and translations of selected sentences [1]

#	Sentence ID	Spanish Transcription	IPA Transcription	English Translation
**1**	ABLANDADA	La patata no está bien ablandada	[la pa'tata no 'esta βjen aβlan'dada]	"The potato is not soft enough"
**2**	ACAMPADA	Mañana vamos de acampada	[ma'ɲana 'bamos de akam'pada]	"Tomorrow we are going camping"
**3**	BARBAS	Cuando las barbas de tu vecino veas pelar, pon las tuyas a remojar	[kwando las 'barβas de tu βe'θino βeas pelar pon las tujas a remo'χar]	"When your neighbor’s beard you see peeling, put yours to soak"
**4**	BURRO	Burro grande ande o no ande	['bur.o 'gran.de an.de o no 'an.de]	"Big donkey walk or not walk"
**5**	CALLE	De la calle vendrá quien de tu casa te echará	[de la kaje βen'dra kjen de tu kasa te etʃa'ra]	"From outside will come that who will kick you out from your house"
**6**	CARMEN	Carmen baila el mambo	['kar.men 'bai.la el 'mam.bo]	"Carmen dances the mambo"
**7**	DIABLO	Cuando el diablo no sabe qué hacer, con el rabo mata moscas	[kwando el djaβlo no sabe ke aθer kon el raβo mata moskas]	"When the devil does not know what to do, it kills flies with its tail"
**8**	GANGA	Esto es una ganga	['es.to es u.na 'ɣaŋ.ga]	"This is a bargain"
**9**	MANGA	Juan tira de la manga	['xwan 'ti.ra de la 'maŋ.ga]	"Juan pulls the sleeve"
**10**	PERRO	Dame pan y llámame perro	['da.me pan i 'ʎa.ma.me 'pero]	"Give me bread and call me dog"
**11**	PAN VINO	Al pan pan y al vino vino	[al pan pan i al 'βino 'βino]	"To the bread bread and to the wine wine"
**12**	PATATA BLANDA	La patata blanda es buena	[la pa'tata 'blanda es 'βwena]	"The soft potato is good"
**13**	PETACA BLANCA	La petaca blanca es mía	[la pe'taka 'blanka es 'mi.a]	"The white flask is mine"
**14**	PIDIO	No pidas a quien pidió, ni sirvas a quien sirvió	[no 'piðas a kjen piðjo ni sirβas a kjen sirβjo]	"Do not beg the one who begged, nor serve the person who served"
**15**	SOMBRA	El que a buen árbol se arrima, buena sombra le cobija	[el ke a βwen 'arβol se a'rima 'βwena 'sombra le ko'βixa]	"Who leans close to a good tree is sheltered by good shade"
**16**	TOMAS	Tomás tira de la manta	['to'mas 'ti.ra de la 'man.ta]	"Tomás pulls the blanket"

Table S2. Overview of speech assessment tasks focusing on Velopharyngeal Closure, prosody, intonation, and other vocal functions [1]

#	Task	Description
**1**	Velopharyngeal Closure	Listen-and-repeat phrases **1, 2, 6, 9, 10, 11, 12**, and **13** from Table S1 for velopharyngeal closure assessment.
**2**	Prosody	Listen-and-repeat phrases **3** and **7** from Table S1 for prosody evaluation.
**3**	Intonation-Emotion	Listen-and-repeat phrases **4, 5, 8, 14, 15,** and **16** from Table S1 with emphasis on capitalized words for intonation-emotion assessment.

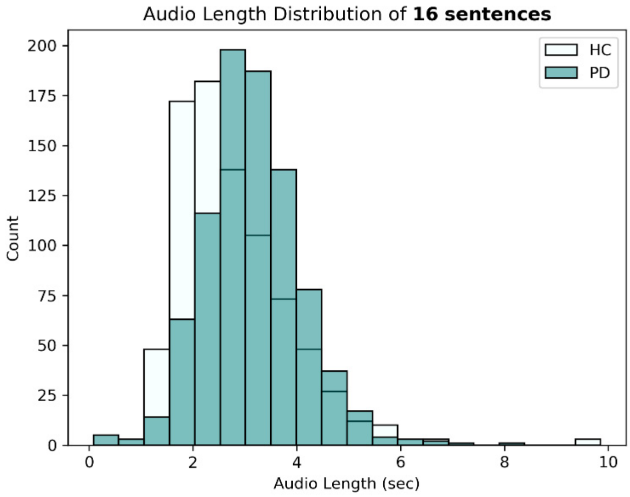

Figure S1. Histogram showing the distribution of audio lengths for the tasks across the groups: HC and PD.

Section S1

eXtreme Gradient Boosting (XGBoost)

XGBoost (eXtreme Gradient Boosting) [2] is a highly efficient, scalable, and flexible machine learning algorithm, well-suited for structured data classification and regression tasks. It operates as an ensemble learning method, building multiple decision trees sequentially, where each new tree corrects the errors of previous ones. This iterative boosting approach optimizes the model by minimizing an objective function that balances both accuracy and model complexity, helping to avoid overfitting while maintaining predictive power [3]. XGBoost uses gradient descent on the residuals (differences between predicted and actual values) of each tree to improve accuracy iteratively. Additionally, it employs techniques such as regularization, handling of missing values, and efficient memory usage, making it particularly effective for high-dimensional data with potentially noisy features, as is often the case in speech feature analysis. Its effectiveness and interpretability have made it a popular choice in various fields, from bioinformatics to financial modeling, and, as in this study, as a baseline for comparison against deep learning approaches in speech classification tasks.

We validated our approach using an XGBoost classifier as baseline model, with hyperparameters optimized through grid search cross-validation. The optimization process explored three key parameters: maximum tree depth (3, 5, 7), learning rate (0.01, 0.1, 0.2), and number of estimators (50, 100, 200).

Table S3. Overview of acoustic features and their associated functionals in eGeMAPS, totaling 88 features [4].

Acoustic Features (Quantity)		Notes
**Frequency (8)**	Pitch (F0)	Logarithmic Fundamental Frequency
	Jitter	Deviations in individual consecutive F0 period lengths
	Formant 1–3 frequency (3)	Center frequency of 1–3 formant
	Formant 1–3 bandwidth (3)	Bandwidth of 1–3 formant
**Energy (4)**	Shimmer	Difference of the peak amplitudes of consecutive F0 periods
	Loudness	Estimate of perceived signal intensity from an auditory spectrum
	HNR	Relation of energy in harmonic components to energy in noise-like components
	Equivalent sound level	Sound level for the noise environment
**Spectral (14)**	Alpha Ratio	Spectral balance between the low-frequency and high-frequency components
	Hammarberg Index	Height of the highest peak in the cepstrum relative to the surrounding peaks
	Spectral Slope (0–500/500–1500) (2)	Rate of change of the spectral energy distribution across different frequencies
	Formant 1–3 relative energy (3)	Ratio of the energy of the spectral harmonic peak at the formant's center
	Harmonic difference H1-H2	Ratio of energy between harmonics
	Harmonic difference H1-A3	Mel-Frequency Cepstral Coefficients
	MFCC 1–4 (4)	Difference of the spectra of two consecutive frames
	Spectral flux	
**Temporal (4)**	Rate of loudness peaks	The number of loudness peaks per second
	Rate of voiced segments	The number of continuous voiced regions per second
	Length of voiced segments	Voiced regions are defined as F0 > 0
	Length of unvoiced segments	Voiced regions are defined as F0 = = 0

Functionals	Measurement
Arithmetic Mean	Average of a set of feature values
Standard Deviation	Variability of a set of feature values
Coefficient of variation	Relative variability of a feature by expressing it as a percentage of the mean
20th/80th percentile	Values at the 20th and 80th percentiles.
Range of 20th ~80th percentile	Range of 20th to 80th percentile
Rising/Falling slope	Slope of rising/falling slope

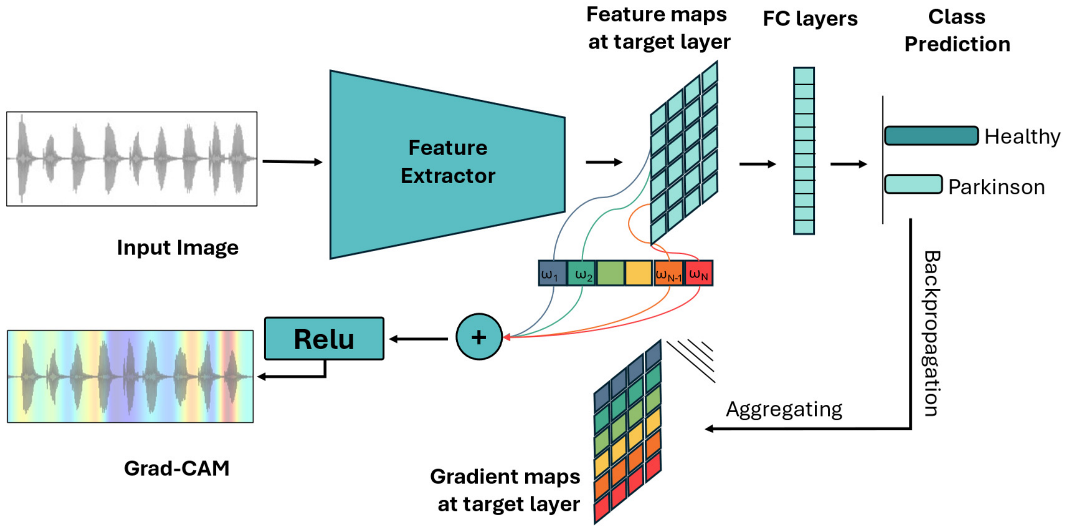

Figure S2. Grad-CAM architecture employed for visual explanation.

**Model**	MFCCs Features	eGeMAPS Features
XGBoost	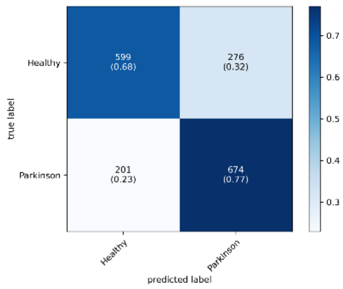	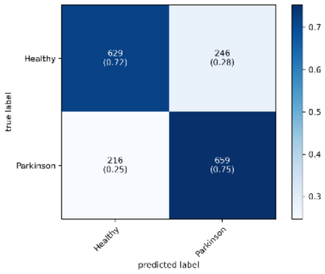
MLP	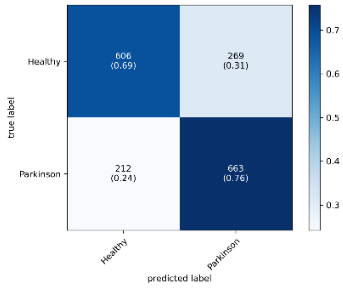	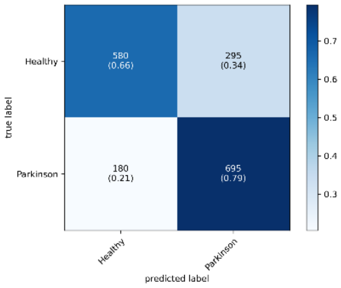

Figure S3. The cumulative confusion matrices show the performance of baseline model across five folds of cross-validation on the dataset of sentences.

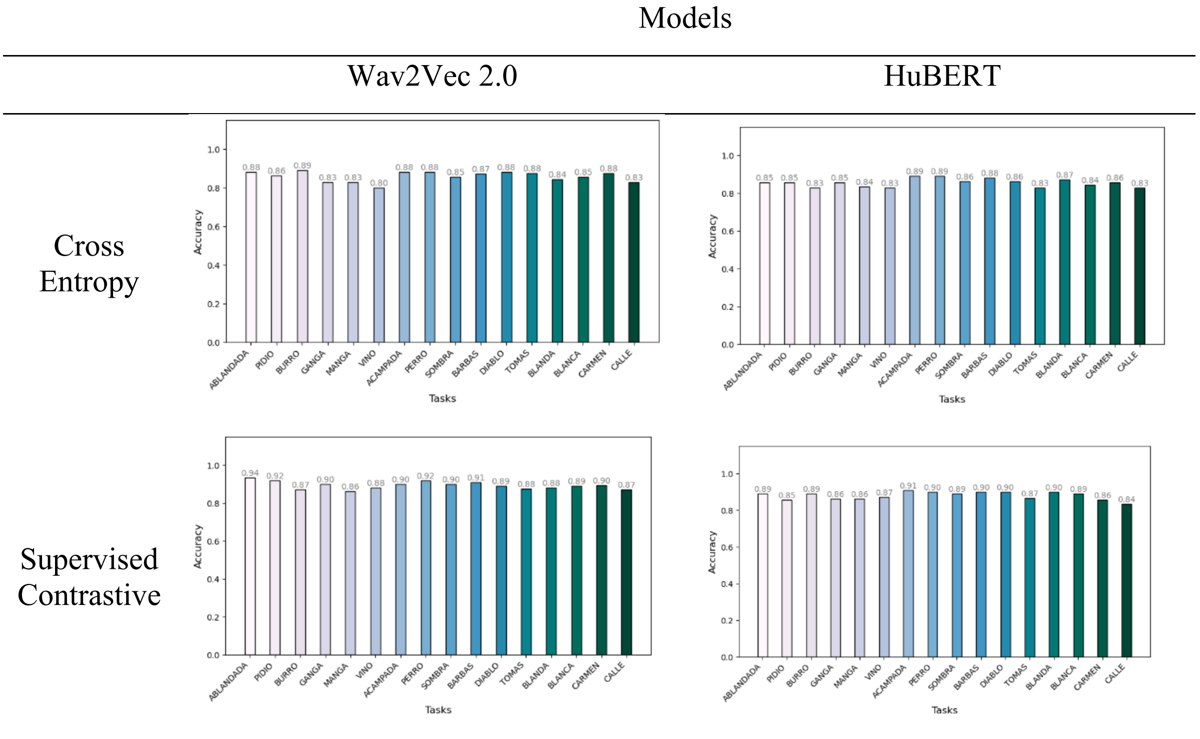

Figure S4. Accuracy comparison across various sentences for Wav2Vec 2.0 and HuBERT models using Cross Entropy and Supervised Contrastive loss functions. The top row displays the performance with Cross Entropy loss, and the bottom row shows the results with Supervised Contrastive loss. Each bar represents the accuracy for a specific sentence, demonstrating the effectiveness of each model and loss function combination.

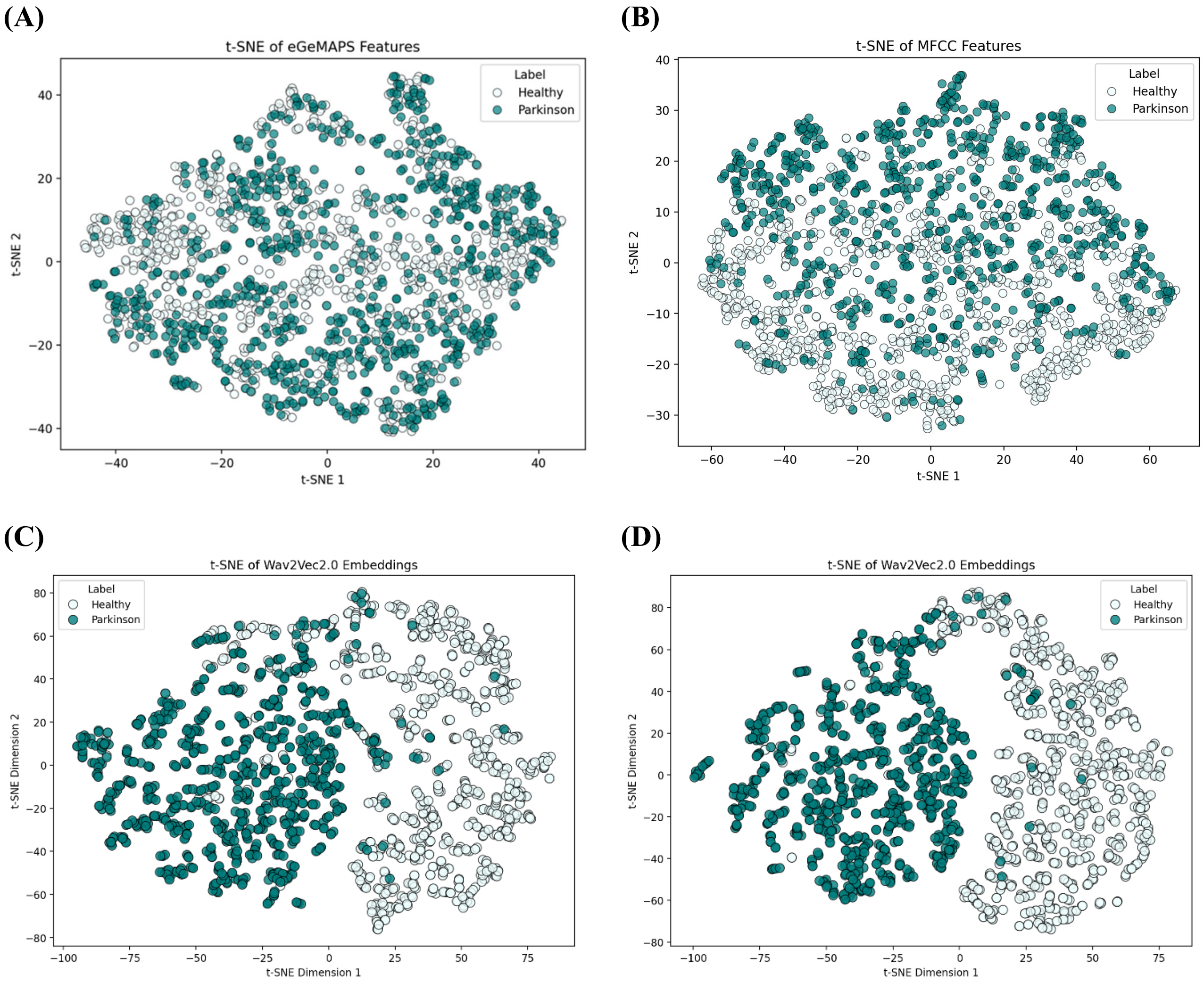

Figure S5. t-SNE Visualizations of Feature Representations for Parkinson's Detection. t-SNE plots show the distribution of Healthy (gray) and Parkinson's (teal) speech samples using four feature types: (A) eGeMAPS, (B) MFCC, (C) raw Wav2Vec 2.0 embeddings with CE, and (D) Wav2Vec 2.0 embeddings fine-tuned with SupCon loss.

Table S4. Classification performance of Wav2Vec 2.0 and HuBERT with Cross Entropy using different layer as feature extractor, showing accuracy, precision, recall, F1, AUC, and specificity with standard deviations.

Metric / Model	Layer for feature ex.	AC[%]	F1[%]	P[%]	SN[%]	SP[%]
**Wav2Vec 2.0 + CE**						
	Layer 1	80.11	80.29	79.89	81.26	78.97
		(±2.40)	(±2.41)	(±4.67)	(±6.51)	(±7.55)
	Layer 6	84.29	84.90	81.98	88.46	80.11
		(±1.58)	(±1.53)	(±4.09)	(±5.50)	(±6.41)
	Layer 12	85.89	86.86	82.02	92.69	79.09
		(±4.27)	(±3.42)	(±5.89)	(±4.30)	(±9.09)
**HuBERT+ CE**						
	Layer 1	80.51	80.51	81.01	81.14	79.89
		(±4.21)	(±24)	(±24)	(±24)	(±10.96)
	Layer 6	83.71	83.92	83.04	85.03	82.40
		(±2.10)	(±2.04)	(±3.70)	(±4.13)	(±4.61)
	Layer 12	85.49	86.27	82.10	91.31	79.66
		(±2.41)	(±2.26)	(±24)	(±5.86)	(±6.88)

The Table S4 presents a comparison of the classification performance of models with Cross Entropy objective using different layers as feature extractors. For both architectures, there is a clear trend of improved performance as we move from down to top layer, particularly in accuracy and F1 scores. Wav2Vec 2.0 achieves its best performance at Layer 12 with an accuracy of 85.89% (±4.27) and F1 score of 86.86% (±3.42), while HuBERT shows comparable results with 85.49% (±2.41) accuracy and 86.27% (±2.26) F1 score at Layer 12. This improvement in higher layers can be attributed to the models' hierarchical feature learning capability, where deeper layers capture more complex and task-relevant representations. Notably, both models show particularly strong sensitivity (SN) at Layer 12, with Wav2Vec 2.0 reaching 92.69% (±4.30) and HuBERT achieving 91.31% (±5.86), suggesting excellent performance in correctly identifying positive cases. The relatively higher standard deviations in specificity (SP) metrics across all layers indicate more variability in identifying positive cases, which may be attributed to the inherent complexity of the data distribution and classification task.

Table S5. Classification performance of Wav2Vec 2.0 and HuBERT with SupCon at with and without scaling hard negatives, and across two different projection sizes (32 and 64), showing accuracy, precision, recall, F1, AUC, and specificity with standard deviations.

Model \ Metric	Scaling Hard Negatives	AC[%]	F1[%]	P[%]	SN[%]	SP[%]
**Wav2Vec 2.0 + SupCon**						
Projection size 32	✔	89.43 (±2.31)	90.00 (±2.35)	85.31 (±2.77)	95.43 (±4.83)	83.43 (±4.00)
	×	88.51 (±1.89)	89.15 (±1.57)	84.75 (±3.40)	94.17 (±2.47)	82.86 (±4.87)
Projection size 64	✔	89.09 (±1.75)	89.63 (±1.53)	85.65 (±3.47)	94.17 (±3.29)	84.00 (±4.83)
	×	87.60 (±2.90)	88.45 (±2.29)	83.35 (±4.62)	94.51 (±3.99)	80.69 (±7.49)
**HuBERT+ SupCon**						
Projection size 32	✔	88.06 (±2.97)	88.99 (±2.48)	83.02 (±4.52)	96.11 (±3.11)	80.00 (±6.60)
	×	88.17 (±2.56)	88.90 (±1.98)	84.34 (±4.70)	94.29 (±3.48)	82.06 (±7.28)
Projection size 64	✔	87.83 (±1.55)	88.72 (±1.38)	82.77 (±2.27)	95.66 (±2.51)	80.00 (±3.38)
	×	87.83 (±1.84)	88.70 (±1.56)	83.01 (±3.34)	95.43 (±3.55)	80.23 (±5.23)

Table S5 compares the performance metrics of models with SupCon, analyzing how scaling hard negatives and different projection sizes (32 and 64) affect various classification metrics. Wav2Vec 2.0 consistently outperforms HuBERT across most metrics, particularly in accuracy (AC), F1-score, and precision (P). The use of scaling hard negatives generally improves performance for both models, with Wav2Vec 2.0 showing more substantial improvements. Notably, the projection size of 32 tends to yield better results than 64 for both models, suggesting that smaller projection dimensions may be more effective for this particular task. The standard deviations indicate relatively stable performance across different runs, with most metrics showing variations of 2-4 percentage points.

Reference

[1] J. Mendes-Laureano *et al.*, "NeuroVoz: a Castillian Spanish corpus of parkinsonian speech," Mar. 06, 2024, *arXiv*: arXiv:2403.02371. doi: 10.48550/arXiv.2403.02371.

[2] T. Chen and C. Guestrin, "XGBoost: A Scalable Tree Boosting System," in *Proceedings of the 22nd ACM SIGKDD International Conference on Knowledge Discovery and Data Mining*, in KDD '16. New York, NY, USA: Association for Computing Machinery, Aug. 2016, pp. 785–794. doi: 10.1145/2939672.2939785.

[3] Department of Production Engineering, Jadavpur University, India, S. Chakraborty, S. Bhattacharya, and Department of Production Engineering, Jadavpur University, India, "Application of XGBoost Algorithm as a Predictive Tool in a CNC Turning Process," *Rep. Mech. Eng.*, vol. 2, no. 2, pp. 190–201, Sep. 2021, doi: 10.31181/rme2001021901b.

[4] S.-M. Jeong, Y.-D. Song, C.-L. Seok, J.-Y. Lee, E. C. Lee, and H.-J. Kim, "Machine learning-based classification of Parkinson's disease using acoustic features: Insights from multilingual speech tasks," *Computers in Biology and Medicine*, vol. 182, p. 109078, Nov. 2024, doi: 10.1016/j.compbiomed.2024.109078.
